# Why do reviewers disagree? Evidence from four funders and 134,000 reviews

**DOI:** 10.1371/journal.pone.0356108

**Published:** 2026-09-23

**Authors:** Jan-Ole Hesselberg, Pål Ulleberg, Øystein Sørensen, Knut Inge Fostervold, Sigrid Hegna Ingvaldsen, Ida Svege

**Affiliations:** 1 Department of Psychology, University of Oslo, Oslo, Norway; 2 The Foundation Dam, Oslo, Norway; 3 NIFU Nordic Institute for Studies of innovation, research and education, Oslo, Norway; University of Gothenburg, SWEDEN

## Abstract

Grant peer review processes are pivotal in allocating substantial research funding, yet concerns about their reliability persist, primarily due to low inter-rater agreement. This study is a registered report that examines factors associated with agreement among peer reviewers in grant evaluations, leveraging data from 134,991 reviews across four Norwegian research funders. Using a cross-classified linear regression model, we explored the relationship between inter-rater agreement and multiple factors, including reviewer similarity, experience, expertise, research area, application characteristics, review depth, and temporal trends. Our analyses revealed several statistically significant associations, but most effects were small in absolute magnitude. Reviewer similarity in gender was associated with lower disagreement. The use of in-depth review procedures, referring to review roles with greater responsibility for evaluating the application, was also associated with slightly lower disagreement. In contrast, expert reviews, defined as assessments conducted by reviewers selected for subject-matter expertise rather than general panel membership, and certain research areas showed modestly higher disagreement. Application amount, reviewer age composition, and temporal trends exhibited limited influence. Only a small share of disagreement stemmed from stable reviewer tendencies (3.1 percent), whereas most variability arose at the application level (24.1 percent) and the residual level (55.2 percent), indicating that disagreement is largely application-specific rather than reviewer-driven. Inter-rater reliability was moderate across funders: average ICCs ranged from 0.50 to 0.65. Our findings only partly support previous research and indicate that disagreement is driven primarily by application-specific ambiguity and inherent variability in human judgment rather than systematic reviewer characteristics or program features. Consequently, interventions targeting reviewer behaviour alone are unlikely to substantially improve reliability; gains are more likely to come from clearer criteria, more structured review formats, and reducing application-level ambiguity, though such measures are expected to yield only incremental improvements.

## Introduction

### Background

Enormous funds are distributed through grants that rely on peer review processes to select which projects to fund. According to the latest review of grant peer review, more than 95% of academic medical research funding is awarded through peer review processes [1]. In the US, the National Institutes of Health (NIH) invests around USD 50 billion every year in health research alone, about 80% of them through competitive grants [2], and in the EU nearly EUR 100 billion will be distributed through the Horizon Europe initiative [3]. In Horizon 2020, the predecessor of the Horizon Europe initiative, over 20,000 expert reviewers were involved in reviewing applications [4].

However, there is considerable debate about how well peer review predicts the future impact of a study. In the most recent review of the literature, authors conclude that “There is (..) fairly clear evidence that peer review is, at best, a weak predictor of future research performance” [1]. The authors cite several possible reasons why this is the case and highlight the problem of low agreement between reviewers scoring the same application as one of the main reasons.

Disagreement is not necessarily a problem. Reviewers bring different expertise and perspectives, and some disagreement reflects legitimate differences over genuinely debatable proposals; a very high level of agreement could even signal that reviewers are too alike to catch what each other miss [5,6]. Agreement is therefore not sufficient on its own to secure review quality. But there are limits: even if all disagreement were of the desirable kind, too much of it makes the process unreliable, and without adequate reliability the validity of peer review is threatened [7]. To put it as a metaphor: take two readings of the same body temperature moments apart; a thermometer whose readings differ by 0.3 °C can still be trusted, but one whose readings differ by 3 °C cannot, however good the reason for the variation.

Several studies have shown that grant reviews often have poor reliability, and almost never reach what is considered acceptable or “good” reliability, commonly defined as an intraclass correlation of 0.75 [7,8]. In the case of grant peer review, an intraclass correlation of 0.75 can be interpreted to mean that 75% of the variance in the ratings given by peer reviewers is attributable to the applications. Hence 25% are attributable to other factors than the applications, like attributes of the reviewers (for example their scoring styles or their school of thought), or other situational attributes (for example the order the applications are reviewed in or if and how panel discussions are run).

Reported single-rater intraclass correlations are consistently low: from 0.17 to 0.37 in an early study of National Science Foundation reviews [9], 0.26 at the Austrian Science Fund [10] and 0.29 at a Norwegian funder even after an intervention intended to raise agreement. By the Spearman-Brown formula [11], reaching the 0.75 threshold at these values would require between 6 and 15 reviewers per application.

Low levels of agreement means that other factors than the applications matter for the outcome, and in a survey of 10,023 ratings by the Australian Research Council the authors conclude that the decision of whether or not an application was granted, was based substantially on chance [8]. In the same paper, the authors highlight the gravity of the problem and claim that the “most basic, broadly supported, and damning criticism” of grant peer review “is its failure to achieve acceptable levels of agreement among independent assessors”. This problem of low agreement in grant peer review seems to generalize across different academic disciplines [8,10].

Several studies have examined factors associated with reviewer ratings or with agreement [1,8,12,13], typically one or a few at a time. Despite the problem being well documented, few have modelled how multiple factors jointly shape agreement. To our knowledge only the Austrian Science Fund study (23,414 ratings) has done so [10]: modelling the determinants of inter-rater reliability, it found research area to be the main predictor (reliability higher in the humanities than in the biosciences), while requested grant amount had at most a weak effect and applicant gender, reviewer gender, and applicant age showed none.

There will always be significant differences between funders that might affect agreement. Differences in review criteria, review type, reviewer experience, the size of the proposed research projects, the scope of the funding programs, and many other factors might make associations that are found at one funder disappear at other research funders. There is a need for larger studies, including more funders and more explanatory factors to assess the robustness of previous findings and to test other hypothesised associations [1,14].

To make sense of where reviewer disagreement comes from, a useful starting point is Kahneman, Sibony and Sunstein’s three-part taxonomy of noise — the unwanted variability that arises wherever human judgments are made under uncertainty [15]. *Level noise* is the persistent tendency for some judges to score systematically higher or lower than others, regardless of the case in front of them. *Pattern noise* is idiosyncratic to the judge–case combination: the way reviewer A is consistently harsher than peers on quantitative proposals while reviewer B is consistently harsher on qualitative ones. *Occasion noise* is transient and within-reviewer — the variability the same person would show reviewing the same application on a different day. Cutting across this taxonomy is a second distinction, between disagreement that originates in who the reviewers are – the individual level – and disagreement that originates in the review process itself – the structural level.

In this study, we use this taxonomy and data from 134,991 reviews at four different Norwegian research funders, to explore factors associated with agreement in grant peer review. To our knowledge this will be the first study on this topic, that uses data from multiple funders and that has predetermined research questions, methods and code.

### Research questions and predictions

The research questions follow the distinction between individual-level (IL) and structural-level (SL) sources of noise. The two levels interact: reviewers are not assigned at random, so individual attributes such as specialist knowledge reach an application through the funder’s matching procedure. We return to the distinction in the Discussion.

### Individual-level sources

#### IL1 are similarities between reviewers associated with agreement?.

The “matching hypothesis” [8], grounded in the mere-exposure effect [16], predicts that similar reviewers agree more. Evidence is mixed: data from the Frontiers journals show same-gender homophily [17], while studies using data from the Australian Research Council found no support for the hypothesis in terms of gender, academic title or age [8,12,18]. We expected similarity in reviewer characteristics to be associated with higher agreement.

#### IL2 is reviewer experience of reviewing positively associated with agreement?.

Previous research has shown that reviewers who rate many applications tend to be both stricter and more reliable than those who rate few [8,12,19]. Seeber and colleagues, analysing over 50,000 European Union applications from 2014–2018, found that experience on prior calls of a specific scheme “significantly reduces disagreement” [20]. We expected the reviewer’s experience to be positively associated with levels of agreement.

#### IL3 is the reviewer’s level of specialist knowledge associated with agreement?.

Some funders use hand-picked external reviewers who receive applications within their field of expertise, whereas panel reviewers more often score applications outside theirs. Reviewers in the latter setting are likely to review some applications that are outside their field of expertise. We expected the hand-picked expert reviewers to agree more, than panel reviewers.

### Structural-level sources

#### SL1 is the application amount associated with agreement?.

Larger amounts likely attract more experienced reviewers, longer review time, and panel calibration; smaller amounts likely produce shorter applications, narrower focus, and greater quality variability. Both could plausibly raise agreement, for opposite reasons. Mutz and colleagues found a statistically significant but practically negligible positive association [10], which we expected to replicate.

#### SL2 is the variability of project size within funding programs associated with agreement?.

Programmes with a wider range of accepted amounts contain more heterogeneous projects, which should make quality differences easier to detect. We expected greater within-programme variability to be associated with higher agreement.

#### SL3 is the review type associated with agreement?.

There are differences in the responsibilities of reviewers assessing the same application. Some funders use primary and secondary reviewers, where the primary reviewers typically are expected to do an in-depth review. This is also the case for external, hand-picked experts.. We expected in-depth reviews to be associated with higher agreement than less-in-depth ones.

#### SL4 is the research area associated with agreement?.

Reviewer scores vary considerably by funding programme and research topic [21]. Mutz and colleagues’ analysis of 23,414 Austrian Science Fund ratings reported the lowest disagreement in the humanities and the highest in the biosciences [10]. This might seem counterintuitive on its face, but is consistent with the logic of interrater-reliability coefficients such as the ICC, which rise when between-application variance is large relative to within-application variance. We expected humanities and social-science applications to show lower disagreement than those in technical, natural, medical, or bio sciences.

#### SL5 is there a trend over time towards higher agreement in grant peer review?.

Increased attention to review-process design, instructions, and reviewer training could raise agreement; turnover, changing criteria, and budget cuts could lower it. Mutz and colleagues found no systematic trend across 1999–2009 [10], which we expected to replicate.

## Methods

### Data

#### Data collection procedures.

Data was collected directly from four different Norwegian funders:

1. The Foundation Dam (Dam). Data retrieved from the grant management system Insights Grants by JOH June 4th 2024.
2. The Kavli Trust (KT). Data retrieved from the application system SurveyMonkey Apply by JOH June 4th 2024.
3. The Norwegian Cancer Society (NCS). Data was retrieved by staff at the NCS from their grant management system Insights Grants and emailed to JOH January 27th 2024.
4. The Norwegian Research Council (NRC). Data was retrieved by staff at the NRC from their grant management system and emailed to JOH January 27th 2024.

One of the authors (JOH) had access to the complete data set. The data set does not contain information that could be used to identify individual participants directly, but contains information that in combination with other public information might make this possible. The use of the data has been approved by the Norwegian Agency for Shared Services in Education and Research (application number: 764354).

The data was partially observed before the analyses was performed to retrieve basic information on the contents, but key variables were not observed, and no statistical analysis was performed prior publication of the protocol. This corresponds to a level 2 bias control, according to the Peer Community Inn Registered Report standards [22].

### Sample

The primary data set, before any exclusion criteria were implemented, consisted of 134,991 reviews of 28,573 applications, done by a total of 6,014 reviewers, between 2013 and 2024. All applications were reviewed on a scale from 1 (poor) to 7 (excellent). From 2019 three of the funders (NRC, Dam and the Kavli Trust) all use the same set of criteria; Excellence, Impact and Implementation. All funders match the applications to the expertise of their reviewers or groups of reviewers. Funders differ in matching granularity, pool size, and whether a coordinator or an algorithm makes the assignment, but matching (not randomisation) is universal. Table 1 shows the distribution of data between the four funders.

**Table 1:** Contents of the primary data set.

*Funder*	*years, range*	*No. of programs*	*No. of applications*	*No. of reviews*	*No. of reviewers*
Dam	2020-2024	1	1,735	8,680	50
KT	2017-2024	1	297	1,339	12
NCS	2017-2023	1	1,105	6,659	115
NRC	2013-2023	93	25,436	118,313	5,837
**Total**	**2013-2024**	**96**	**28,573**	**134,991**	**6,014**

The codebook describing the primary data set in detail can be found in the supplementary information (see S1 Table).

### Data structure

**Fig 1:**
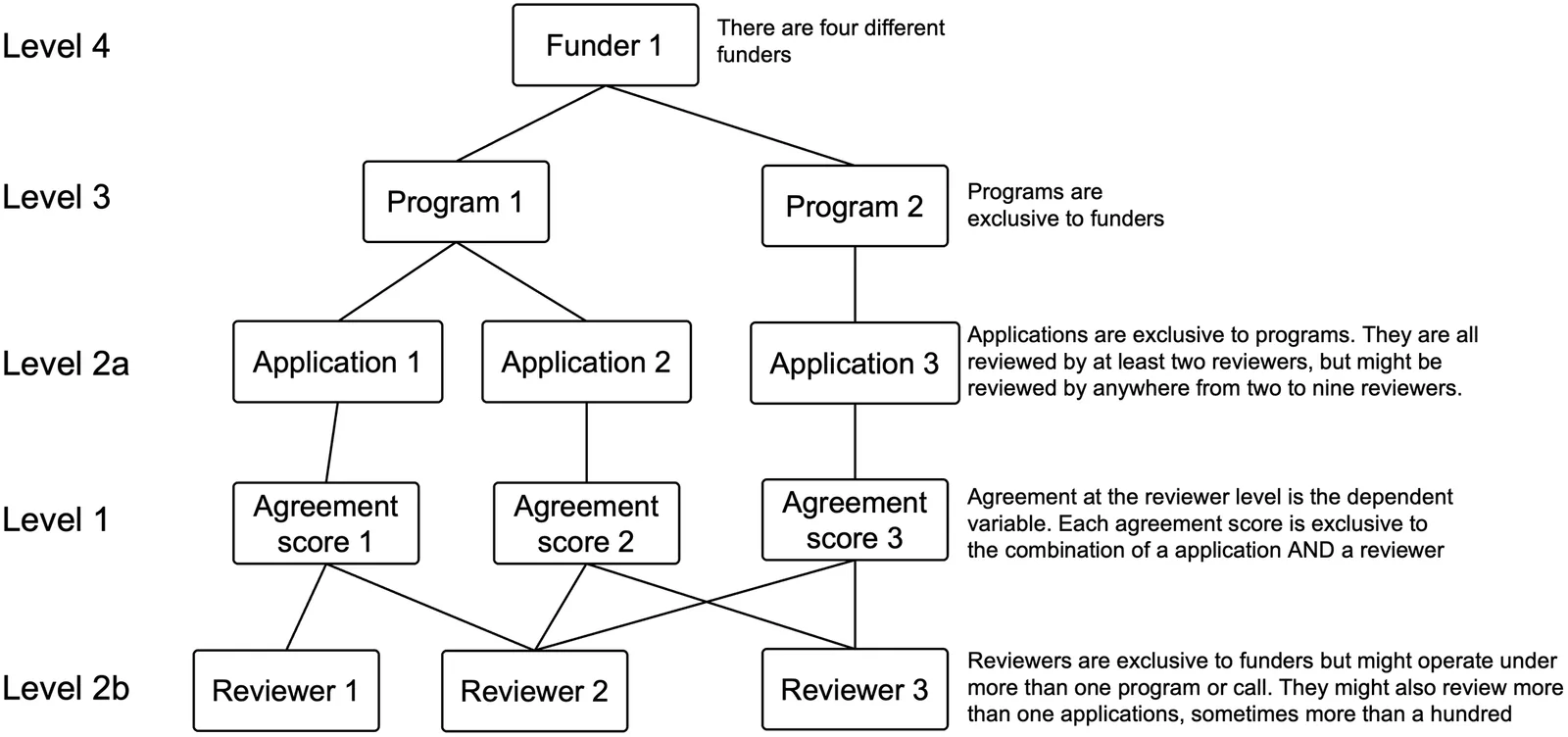
Data structure.

The data has a cross-classified structure with the following important properties at different levels (Fig 1):

- **Level 4 – Funder.** Four different funders with different programs.
- **Level 3 – Programs.** The funding programs are exclusive to funders. The Norwegian Research Council is the only funder with more than one program.
- **Level 2a - Applications** are exclusive to programs. They were all reviewed by anywhere from two to nine reviewers.
- **Level 1 – Agreement score.** The agreement between reviewers assessing the same application.
- **Level 2b - Reviewers** are exclusive to funders but might operate under more than one program. In addition, they might review more than one application, sometimes more than a hundred.

### Variables

#### Response variable.

The response variable is agreement between reviewers of the same review type assessing the same application. Hence, only applications with two or more independent review scores of a particular review type (external expert, panel member or depth reviewer) were included in the analyses.

Agreement was assessed by using the Average Deviation (AD) index. The AD index shows how much a reviewer’s assessment of an application deviates from the mean of the individual scores of the application. When the average AD index for an application is zero, the raters are in complete agreement, and higher scores indicate more disagreement.

The following formula was used:


$$\begin{array}{c} {Disagreement}_{ij}=\left|{Score}_{ij}-\frac{\text{1}}{n_{i}}\sum_{j=\text{1}}^{n_{i}}{Score}_{ij}\right| \end{array}$$

In the formula, *i* represents the application and *j* the reviewer. Hence *Score*_*ij*_ represents the reviewer’s score of an application. *n* stands for the number of reviewers (*j*) assessing a specific application of a specific review type (*i*)

In this study, the reviewers score on an application (*Score*_*ij*_) was either an overall score set by the reviewer or, where this was not available, we calculated an aggregate score based on the reviewers criteria scores. All applications and review criteria were scored on a scale from 1 (poor) to 7 (excellent). The minimum average AD index for an application is 0 and the maximum is 3.0.

We chose to use the AD index as our primary indicator of inter-rater (dis)agreement, with intraclass correlation coefficients (ICC) reported in exploratory analyses for benchmarking against prior literature. The AD index quantifies disagreement in the original 1–7 scale units, making it directly interpretable and supporting effect-size statements of the form “a change of X units, equivalent to Y percent of the mean.” It does not require a specified null distribution and is therefore robust to assumptions about reviewer calibration [23,24]. ICC, by contrast, is conceptually a reliability coefficient — the proportion of total score variance attributable to differences between applications — rather than an agreement coefficient per se; the two quantities can diverge substantially, particularly when between-application variance is compressed (range restriction; see Discussion). The AD index has been used in earlier studies of grant peer-review agreement [25,26] and simulation research has demonstrated that the AD index performs favourably when compared with other inter-rater agreement indices [27–29].

#### Explanatory variables.

We produced nine explanatory variables that reflect different attributes of the funder, programs, applicant, application, reviewer and the review. For an overview of the explanatory variables, their coding and the predicted associations see Table 2. The numbering of the explanatory variables corresponds with the numbering of the research questions. The steps taken to produce the explanatory variables are described in detail in the attached R script (see S2 File).

**Table 2:** Explanatory variables, coding and predicted associations.

Variable	Coding	Predicted association
**IL1a Reviewer similarity, gender**	Proportion of reviewers of same gender, 0.5–1.0	**Negative association** Higher proportion = Less disagreement
**IL1b Reviewer similarity, age**	Standard deviation (SD) of reviewer age per application (*z*-standardized)	**Negative association** Increased SD = Less disagreement
**IL2 Proportion of young reviewers**	Proportion of reviewers that are among the 20 percent youngest reviewers in the data set	**Positive association** Higher proportion = More disagreement
IL3 Reviewer specialist knowledge	1 = specialist 0 = non-specialist	Negative association Less disagreement for specialist reviews than for non-specialist reviews
**SL1 Application amount**	Requested grant amount in million NOK (*z*-standardized)	**Negative association** Higher application amount = Less disagreementAssociation will probably not be of practical importance
**SL2 Variability in application amount of program**	Coefficient of variation (CV): SD of applied amount in program divided by the program mean (*z*-standardized)	**Negative association** Higher variability = Less disagreement
**SL3 Review depth**	1 = in-depth review 0 = panel member (less depth)	**Negative association** Less disagreement for in-depth reviews than for panel member reviews
**SL4 Research area**	3a Humanities 1/0 3b Social sciences 1/0 3c Technology 1/0 3d Med and health 1/0 3e Math and natural 1/0 3f Agriculture 1/0 3g Other 1/0	**Associations:** Humanities < Technology Humanities < Math & natural sci. Humanities < Medicine and health Social sci. < Technology Social sci. < Math & natural sci. Social sci. < Medicine and health
**SL5 Year of submission**	Year of submission (2013–2024; mean-centered)	**No association** There will not be a significant trend towards more or less disagreement over the years

Note. A total of 15 predictions were made. IL = Individual level factors. SL = System-level factors.

### Individual level factors

#### IL1a proportion of reviewers of same gender.

The proportion of reviewers of the same gender was calculated for each review of a particular review type (external expert, panel member or depth reviewer) per application. For example: If two out of three reviewers were male, the value returned was 0.67. The same value would have been returned if two out of three were female.

#### IL1b standard deviation of reviewer age per application.

The standard deviation of reviewer age was calculated for each application and review type (external expert, panel member, or depth reviewer), reflecting the age diversity among reviewers assessing the same application. To improve numerical stability and comparability, this variable was *z*-standardized (mean = 0, SD = 1) prior to analysis.

#### IL2 proportion of young reviewers.

As we do not know how many reviews the reviewers have done before the first review in the dataset, we do not have a reliable and direct way of assessing the reviewers’ level of reviewing experience. We considered each reviewer’s cumulative number of reviews within the data set as a more direct measure, but set it aside because it reflects only reviewing recorded within our observation window and is not comparable across funders whose data sets differ in span and volume. As a proxy for the variability in reviewer experience within the review of an application, we will calculate the proportion of reviewers that are among the 20 percent youngest reviewers in the data set, at the time of the review. For example: If two out of three reviewers are among the 20 percent youngest, the value returned will be 0.67.

#### IL3 reviewer specialist knowledge.

External reviewers are usually experts within the topic of the application. This variable will be coded TRUE if the review type is External expert and FALSE if the review type is Panel member or Depth reviewer.

### System level factors

#### SL1 application amount.

The requested grant sum of the application (in million Norwegian kroner, NOK) was used as a continuous predictor. To ensure numerical stability and comparability of effect sizes, the variable was *z*-standardized (mean = 0, SD = 1) prior to analysis.

#### SL2 coefficient of variation in application amount of program.

To capture the variability in application amounts within each funding program, we calculated the coefficient of variation (CV) by dividing the standard deviation of application amount (in million NOK) by the program mean. The resulting measure was *z*-standardized (mean = 0, SD = 1) before inclusion in the model.

#### SL3 review depth.

There are three review types in the data set: External expert, Panel member or Depth reviewer. External experts and Depth reviewers are both performing more in-depth reviews than the panel members. This variable will be coded TRUE if the review type is External expert or Depth review, and FALSE if the review type is Panel member.

#### SL4 research area.

The research area of each application. Seven categories are used:

a) Humanities,
b) Social sciences,
c) Technology,
d) Medicine and health,
e) Mathematics and natural sciences,
f) Agriculture and fish,
g) Other

Each of the categories were coded as a logical variable. It is possible for an application to fall under more than one category. All applications from the Cancer society, the Kavli Trust and the Foundation Dam, fall only under the “Medicine and health” category.

#### SL5 year of submission.

The year in which the application was submitted (2013–2024) was included to assess potential time trends in reviewer agreement. The variable was mean-centered to improve model interpretability.

### Analysis plan

#### Statistical models.

To test the hypotheses, we used a cross-classified linear regression model. Similar approaches have been used by Jayasinghe and colleagues [12] and Seeber and colleagues [26]. This approach accounts for the fact that the disagreement scores (the response variable) on the one hand are nested in applications within programs within funders, and on the other hand in reviewers across applications and programs. The method also considers that the relationship between the explanatory variables and the response variable (disagreement) may be influenced by unobserved variables impacting both. Since reviewers can evaluate multiple applications, the structure is not hierarchical but cross-classified.

Our preregistered model estimated effects of each research area relative to a reference category, but this specification could not directly test the hypotheses concerning contrasts *between* specific research areas. To address this limitation, we conducted *post hoc* pairwise contrasts between all research areas using the fitted multilevel model. These contrasts provide adjusted comparisons across disciplines and form the basis for the cross-field differences reported in the Results section. All reported differences between research areas therefore originate from these post hoc contrasts rather than from the raw regression coefficients.

The analyses were performed using R 3.6.0 + , RStudio version 2024.04.2 + 764 [30] and the lme4 package version 1.1–35.5 [31].

#### Inference criteria.

We used the standard *p* < .05 criteria for determining if the linear regression coefficients for the hypothesized factors had an effect on the agreement between reviewers. For interpreting effect sizes we used the estimated regression coefficients and their associated 95% confidence intervals.

We expected that some statistically significant results would have little practical significance, but we did not predetermine criteria for inferring the practical significance for the hypothesized factors. The main reason was that the practical significance will vary with who the end user of the results is. A weak association might be of importance to the funder and have even greater implications for the society as a whole but have a negligible impact on the review of individual applications. Additionally, we are not aware of reliable ways to determine the level for practical significance of differences in agreement, given these different end users’ perspectives. For this reason, we decided in advance of the analyses to leave the reflections on practical significance to the discussion of the results.

#### Data exclusion.

In accordance with the prespecified data exclusion criteria, we removed the following data in the following order before analysis:

The following entries will be removed in the following order before analysis:

1. **Unlikely reviewer ages.** Entries of reviewer ages below 21 and above 100 were removed.
2. **Duplicate entries**.
3. **Missing review scores.** Rows where review scores were missing were removed.
4. **Impossible review scores**. Rows containing review scores below the minimum score of 1 and above the maximum score of 7 were removed.
5. **Missing application amounts.** Rows with missing application amounts were removed.
6. **Unlikely application amounts**. Rows containing application amounts that are more than ten times higher than the average amount of the program were removed.
7. All rows with **less than two reviews** of a particular review type per application. At least two reviewers are needed to calculate the response variable.
8. All rows related to **programs with less than 20 applications.** As expected, some programs had very few applications. These programs are likely to have some attributes that make the applications in them especially hard to compare with other applications. In addition, as one of the explanatory variables is variability in application amount of programs, we will need some applications to calculate a reliable value. Therefore, we only included programs with 20 or more applications.

#### Missing data.

In accordance with the prespecified protocol we excluded all rows with missing values to ensure the integrity and interpretability of the results. The cross-classified linear regression model benefits from complete data for all variables to accurately estimate parameters and account for the nested and cross-classified structure of the data. Excluding rows with missing values helps maintain consistency in the dataset and prevents potential confounding due to incomplete information. This approach is particularly important given the complexity of our model, which seeks to disentangle multiple levels of relationships and account for unobserved variables.

#### Sensitivity analyses.

We explored the effect of removing missing data by doing regression analyses of each explanatory variable separately and comparing the results to the main analysis.

#### Exploratory analysis.

We explored possible differences between the funders by performing an ANOVA and visualizing the differences between funders using a box plot. Additionally, we calculated the intraclass correlations (ICC (1,1)) per funder for the two most common review types (panel member and depth reviewer) combined and used the average number of reviews per funder to calculate the average measure intraclass correlation (ICC (1,k)).

#### Codebook and analysis scripts.

The codebook describing the contents of the primary data set is included in the supplementary materials (see S1 Table). Two R scripts are used in this study, a preprocessing script (see S1 File) and the analysis script (see S2 File). The preprocessing script merges the data from the four funders, produces the explanatory variables based on personal data, removes personal data and exports the primary data set for analysis [32]. The analysis script produces the remaining explanatory variables and runs the analysis described above.

#### Deviations from the registered report protocol.

Our analysis plan was preregistered and approved at Stage 1 [33]. A few minor adjustments were made after preregistration to improve model specification, clarity, and numerical stability:

1. Relabelling of explanatory variables. To make the conceptual framework requested in review explicit, the eight research questions and their explanatory variables are regrouped into individual-level sources (IL1 = EV1 in the protocol, IL2 = EV2, IL3 = EV7) and structural sources (SL1 = EV4, SL2 = EV5, SL3 = EV6, SL4 = EV3, SL5 = EV8). Operationalisations, hypotheses, and analyses are unchanged.
2. Three continuous explanatory variables (IL1b, SL1, and SL2) were *z*-standardized (mean = 0, SD = 1), and the year of submission (SL5) was mean-centered. These transformations were introduced to enhance numerical stability and facilitate comparison of regression coefficients; they do not alter the direction or substantive interpretation of the results.
3. A coding error was identified in the preregistered R script, where the hierarchical nesting structure of the random effects was inverted. In the final model, the correct hierarchy was specified as applications nested within programs nested within funders, and reviewers nested within funders. This correction improves the conceptual and statistical accuracy of the model but does not materially affect the substantive conclusions.
4. We clarified the conceptual interpretation of the outcome measure. The Average Deviation (AD) index quantifies **disagreement** rather than agreement among reviewers. To reflect this, all references to the outcome have been rephrased in terms of disagreement, and the predicted directions of association in Table 2 were updated accordingly.
5. The predicted associations for research area (SL4) were made explicit. While the preregistered protocol stated only that disciplinary differences were expected, the final manuscript specifies the hypothesized ordering of fields (e.g., Humanities and Social Sciences with lower disagreement than Medicine and Health and Mathematics and Natural Sciences) in accordance with the original theoretical rationale. In addition, because the preregistered multilevel model estimates each research area only relative to a reference category, it could not directly test these preregistered contrasts. To evaluate the hypotheses as intended, we therefore added post hoc pairwise comparisons between all research areas using the fitted model. These comparisons do not alter the theoretical predictions but provide the appropriate statistical tests for the preregistered hypotheses.

## Results

### Data exclusions and final sample

Following the prespecified exclusion criteria, 37,317 rows (27.6% of the initial dataset) were removed. The largest single reduction (32,137 rows) occurred when removing applications with fewer than two reviews of the same review type (external expert, panel member or depth reviewer). The number of observations excluded at each step is summarized in the supplementary material (see S2 Table).

A total of 97,674 rows were left after data exclusion. Of these, 8,390 rows were removed due to missing data, leaving 89,284 rows for the main analysis. 8,142 of the rows with missing data were from the Norwegian Research Council and the remaining 248 rows were from the Cancer Society.

The final analytical dataset comprised 89,284 reviews of 24,690 applications evaluated by 3,974 reviewers across the four participating funders. Table 3 gives an overview of the data per funder.

**Table 3:** Summary statistics by funder.

Funder	# Programs	# Applications	# Reviewers	# Reviews	Avg. review score	SD review score	Avg. AD index	SD AD index
Cancer Society	1	1,050	110	4,909	4.53	1.11	0.66	0.51
Dam	1	1,716	49	7,859	4.71	0.84	0.54	0.41
Kavli	1	297	12	1,332	4.25	1.64	1.00	0.74
NRC	61	21,627	3,803	75,184	5.03	1.08	0.56	0.46
Total	64	24,690	3,974	89,284	4.96	1.08	0.57	0.47

Note. SD = standard deviation; Avg. = average. Review scores range from 1 (poor) to 7 (excellent). AD index refers to the Average Deviation Index, a measure of reviewer disagreement.

### Main results

Reviewer disagreement, as measured by the AD index, varied systematically with several of the explanatory variables, and a number of these associations were statistically significant. Given the very large number of reviews, however, statistical significance is expected and is not in itself evidence of practical importance. All β values are unstandardized, so to make them interpretable we express each effect relative to the mean AD index, which was 0.570. Consistent with our preregistered inference criteria, we describe the magnitudes here and reserve appraisal of their practical significance for the Discussion.

#### IL1: Reviewer composition.

Proportion of reviewers of the same gender (IL1a) was negatively associated with disagreement (β = −0.022, *p* = 0.015), suggesting that reviews with a higher proportion of same-gender reviewers produced slightly more consistent evaluations. The maximum increase in the proportion of reviewers of the same gender from 0.5 to 1.0 was associated with a 0.01 decrease in the AD index, corresponding to about 2% of the mean AD index.

Standard deviation of reviewer age (IL1b) was positively associated with disagreement (β = 0.007, p < 0.001), indicating that age-diverse reviewer groups tended to generate slightly more variable assessments. A one–standard-deviation increase in age diversity corresponded to roughly a 0.007 increase in the AD index, or about 1% of the mean AD index.

Both results support the prediction.

#### IL2: Proportion of young reviewers.

The proportion of reviewers among the youngest 20% (IL2) showed no statistically significant association with disagreement (β = −0.001, *p* = 0.918), suggesting that the presence of younger reviewers did not contribute meaningfully to variation in scores. The results do not support the prediction.

#### IL3: Reviewer specialist knowledge.

The expert review format (IL3) was associated with greater disagreement (β = 0.088, *p* < .001. Expert reviews were associated with a 0.088 increase in disagreement, about 15% of the mean AD index. This association was in the opposite direction of what was predicted.

#### SL1: Application amount.

The standardized application amount (SL1) was not significantly associated with reviewer disagreement (β = −0.005, *p* = 0.105). This indicates that the size of the requested budget did not systematically affect the level of variability in reviewers’ scores. The results do not support the prediction.

#### SL2: Variability in application amount of program.

The variability in application amount within the funding program (SL2) showed a statistically significant negative association with disagreement (β = −0.013, *p* = 0.014), suggesting that programs with greater dispersion in requested budgets tended to have slightly lower reviewer disagreement. A one–standard-deviation increase in within-program variability corresponded to roughly a 0.013 decrease in the AD index, about 2% of the mean AD index. The results support the prediction.

#### SL3: In-depth review procedure.

In-depth reviews (SL3) were associated with reduced disagreement (β = −0.032, *p* < .001, suggesting that more time and effort spent on the review produced slightly more consistent evaluations. In-depth reviews were associated with a 0.032 decrease in disagreement, about 6% of the mean AD index. This result supported the prediction.

#### SL4: Research area.

Research area (SL4) was associated with systematic but modest differences in reviewer disagreement. The regression coefficients quantify differences relative to the reference category (“Other”). Compared to this reference group, Humanities (SL4a), Social sciences (SL4b), and Technology (SL4c) showed slightly higher disagreement (β = 0.041, *p* < .001; β = 0.018, *p* = .016; β = 0.020, p = .011, respectively), while Mathematics and natural sciences (SL4e) showed lower disagreement (β = –0.031, *p* < .001). Differences for Medicine and health sciences (SL4d) and Agriculture and fish (SL4f) were not statistically significant.

However, our preregistered hypotheses concerned comparisons between specific research areas, not comparisons with the reference category. Because the model coefficients do not directly test these hypotheses, we conducted post hoc pairwise contrasts between all research areas using the fitted model. These contrasts showed that Humanities exhibited the highest disagreement and Mathematics and natural sciences the lowest. The largest contrast – a 0.072 AD-unit difference between Humanities and Mathematics and natural sciences – corresponds to approximately 13% of the mean AD index. None of the preregistered hypotheses predicting lower disagreement in Humanities and Social sciences compared with Technology, Mathematics and natural sciences, and Medicine and health sciences were supported. Three of the contrasts were in the opposite direction of the hypotheses.

**Fig 2:**
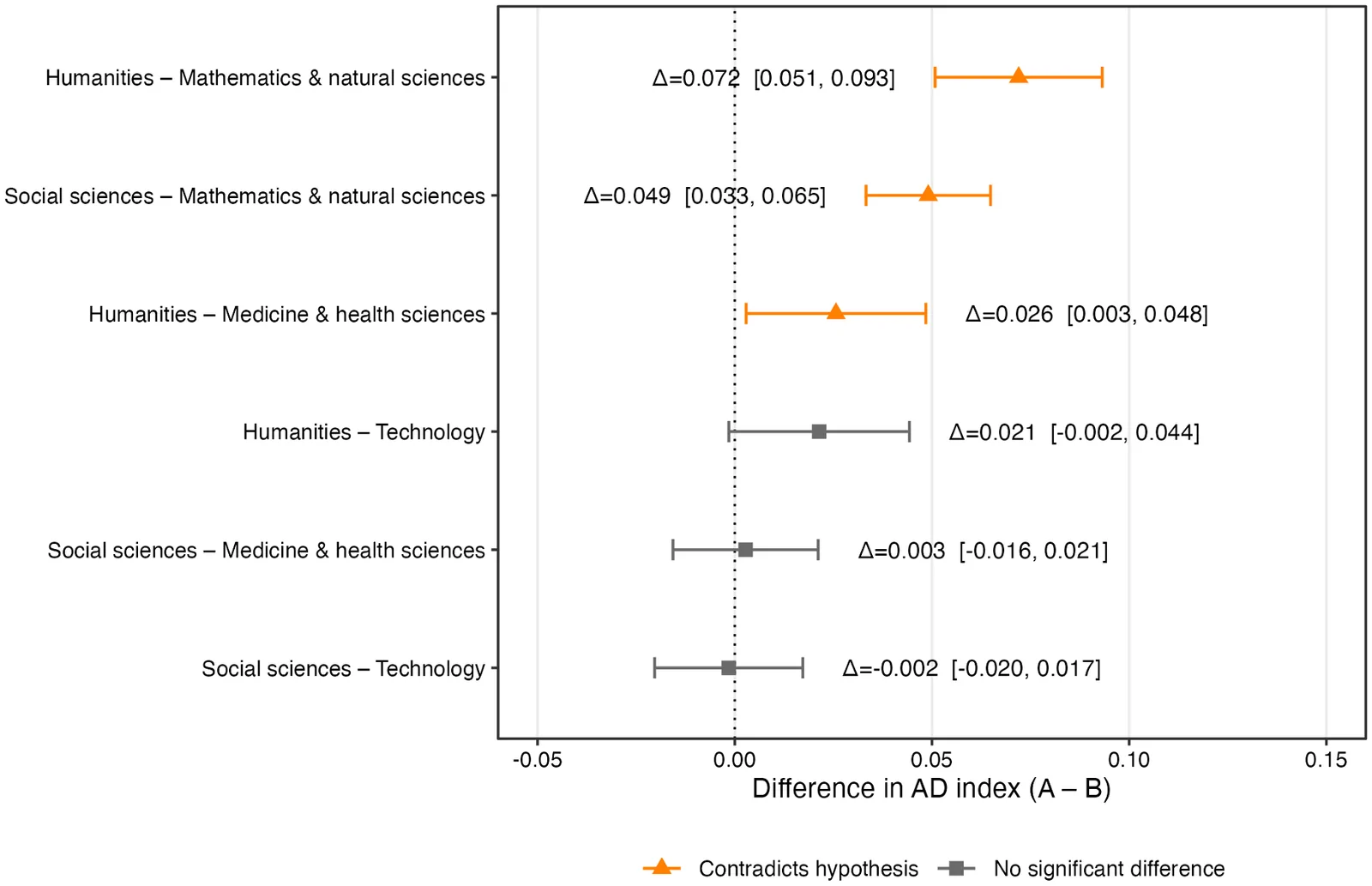
Forest plot of hypothesized contrasts between research areas.

The preregistered contrasts are shown in Fig 2, and all pairwise contrasts are presented in the supplementary materials (S1 Fig).

#### SL5: Year of submission.

The year of submission (SL5) had a small but statistically significant negative effect (β = −0.007, *p* < .001), suggesting that reviewer disagreement has gradually declined over time. A ten-year period was associated with a 0.07 decrease in disagreement, about 12% of the mean AD index. The results do not support the prediction. See S2 Fig for a visualization of the AD index by year.

A full summary of the estimated effects, standard errors, and test statistics is presented in Table 4.

**Table 4:** Result summary.

Variable & Description	β	SE	t-value	p-value	Support for prediction?
**Intercept**	0.720	0.108	6.68	0.007*	
**IL1 Reviewer composition**					
IL1a Proportion same gender	−0.022	0.009	−2.44	0.015*	Yes
IL1b Standard deviation reviewer age ^a^	0.007	0.002	3.67	<.001*	Yes
**IL2 Proportion of young reviewers**	−0.001	0.008	−0.10	0.918	No
**IL3 Expert review**	0.088	0.014	6.33	<.001*	No
**SL1 Application amount (MNOK)** ^a^	−0.005	0.003	−1.62	0.105	No
**SL2 Variability in application amount** ^a^	−0.013	0.005	−2.56	0.014	Yes
**SL3 In-depth review**	−0.032	0.003	−9.19	<.001*	Yes
**SL4 Research area** ^**c**^					
SL4a Humanities	0.041	0.010	4.15	<.001*	No / No / No ^c^
SL4b Social sciences	0.018	0.008	2.40	0.016*	No / No / No ^c^
SL4c Technology	0.020	0.008	2.56	0.011*	
SL4d Medicine and health sciences	0.015	0.008	1.88	0.060	
SL4e Mathematics and natural sciences	−0.031	0.007	−4.43	<.001*	
SL4f Agriculture and fish	−0.010	0.013	−0.77	0.413	
**SL5 Year of submission** ^b^	−0.007	0.002	−4.34	<.001*	No

Note. β = unstandardized regression coefficient unless noted; SE = standard error; CI = 95% confidence interval. Significance codes: * p < 0.05. SL4g Other areas was dropped due to collinearity. ^a^ Standardized regression coefficients ^b^ Centred regression coefficient ^c^ SL4g “Other” was dropped due to collinearity. For SL4 (research area), the regression coefficients represent differences relative to the reference category only and therefore do not directly test the preregistered hypotheses, which concerned contrasts *between* specific research areas (e.g., Humanities vs. Mathematics and natural sciences). These hypotheses were evaluated through post hoc pairwise contrasts based on the fitted model.

### Random effects

Variance partitioning showed that most heterogeneity in reviewer disagreement resides at the residual level (55.2%, SD = 0.381), with a substantial share at the application level (24.1%, SD = 0.211) and the funder level (17.0%, SD = 0.211). Smaller components were attributable to reviewers (3.1%, SD = 0.091) and programs (0.5%, SD = 0.038) (see Table 5).

**Table 5:** Variance Components from the Linear Mixed Model.

Random Effect Level	Variance	SD	Proportion of total variance (%)
Application (nested within program and funder)	0.063	0.211	24.1
Reviewer (nested within funder)	0.008	0.091	3.1
Program (nested within funder)	0.001	0.038	0.5
Funder (highest level)	0.045	0.211	17.0
Residual	0.145	0.381	55.2

Estimated variance, standard deviation (SD) and proportion of total variance of the random effects in the hierarchical model predicting reviewer disagreement.

### Model fit

The model converged successfully using the *bobyqa* optimizer with an increased iteration limit (maxfun = 2e5). No convergence warnings were returned, and the model was not singular. Inspection of alternative specifications confirmed that parameter estimates were stable across models, indicating good numerical performance and robust results.

#### Sensitivity analyses.

Sensitivity analyses, in which each explanatory variable was regressed separately on the outcome using all available data for that variable, generally yielded results consistent with the main analysis (see S3 Table). However, a few deviations emerged.

For IL2 *Proportion of young reviewers*, the main model found no significant association with reviewer disagreement, whereas the sensitivity analysis indicated a small but statistically significant positive association (β = 0.021, p < .001), suggesting slightly more disagreement in panels with more young reviewers.

Similarly, for SL4b *Social sciences*, the main model showed no significant effect, but the sensitivity analysis found a small positive association (β = 0.018, *p* < .001). Conversely, for SL4f *Agriculture and fish*, the main model found no significant association, while the sensitivity analysis detected a small but significant negative association (β = −0.021, *p* = .002), indicating less disagreement in this field.

These discrepancies likely reflect differences in sample composition when including cases with missing data on other predictors. However, the very small effect sizes and low explained variance (all R² ≤ .002) suggest that these associations have limited practical relevance.

#### Exploratory analyses.

**Fig 3:**
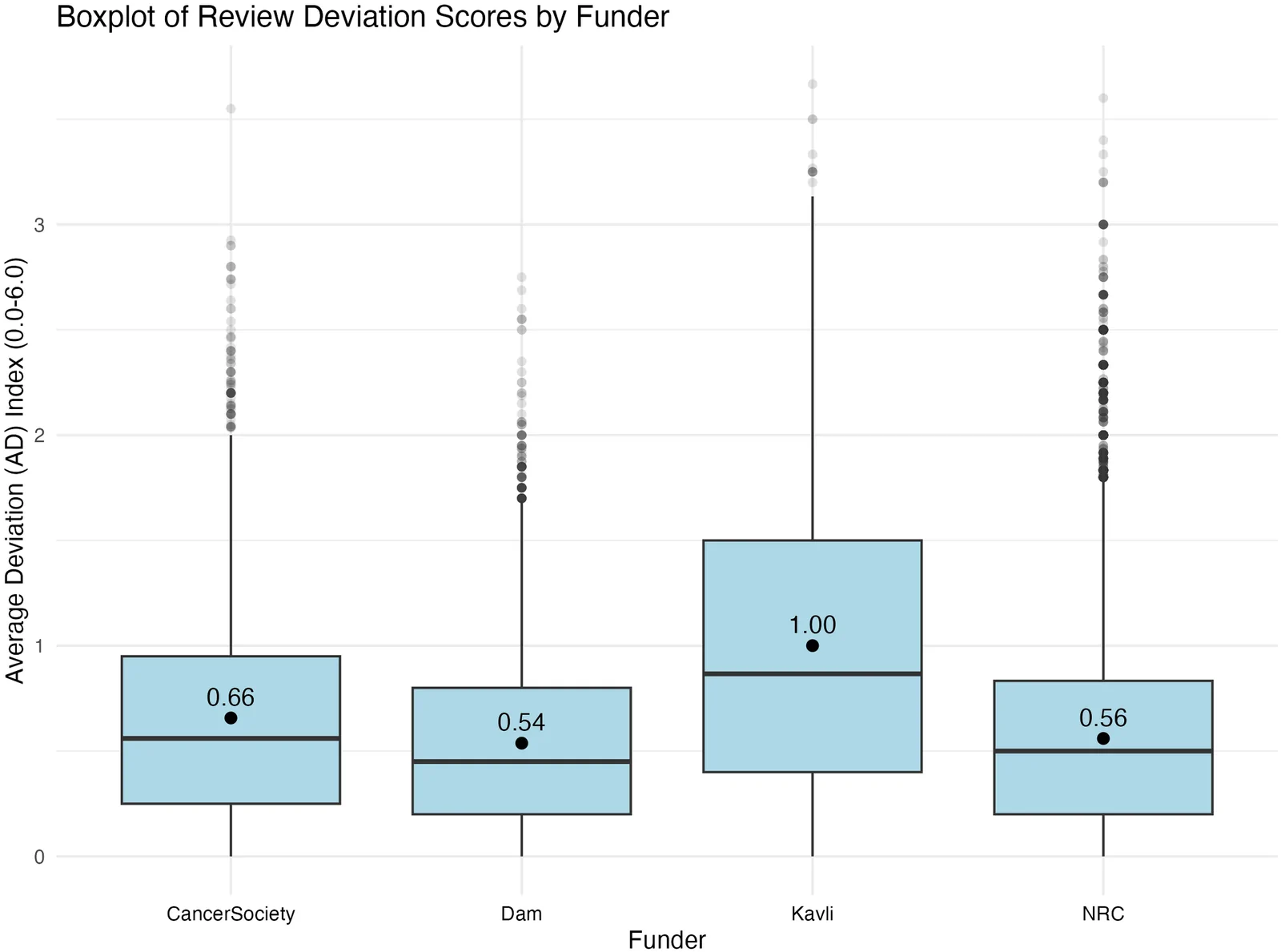
Box plot of average deviation index by funder. Note: The black dots and the numbers in the boxes indicate the mean AD index for each funder. Outliers are shown as semi-transparent points.

Fig 3 shows a boxplot of the absolute deviation scores by funder. The figure highlights that the Foundation Dam had the lowest average deviation from the mean (0.54), while Kavli had the highest (1.00).

The results from the ANOVA test indicated significant differences between funders, *F*(3, 89,280) = 467.3, *p* < .001. Post-hoc Tukey tests showed that all pairwise comparisons between funders were statistically significant, with the largest difference observed between Kavli and Dam (diff = 0.46, 95% CI [0.43, 0.50], *p* < .001).

To further assess the reliability of reviews, we estimated inter-rater reliability using a one-way random-effects intraclass correlation, ICC(1,1), computed separately for each funder via a random-intercept model with application as the target (grouping) and reviewers sampled across applications. As shown in Table 6, single-measure ICCs ranged from 0.18 (Dam) to 0.29 (Cancer Society), indicating low-to-moderate reliability of individual reviews. The reliability of the mean application score, ICC(1,k), increased accordingly to 0.50–0.65 given the observed number of reviewers per application. Using the Spearman–Brown prediction formula [11], we estimated how many independent reviewers would be required for the mean rating to reach ICC ≥ 0.75, a commonly used threshold for “good” reliability [7,11].

**Table 6:** Intraclass correlations by funder.

Funder	# Reviews	# Applications	Avg. # Reviews	ICC(1,1)	ICC(1,k)	# reviewers needed for ICC(1,k) ≥0.75
Cancer Society	4,909	1,050	4.68	0.29	0.65	8
Dam	7,859	1,716	4.58	0.18	0.50	14
Kavli	1,332	297	4.48	0.26	0.61	9
NRC	75,184	21,627	3.48	0.23	0.50	11

Note. ICC(1,1) is the reliability of a single review when reviewers are randomly sampled; ICC(1,k) is the reliability of the mean of k independent reviews (here, k = the funder-specific average number of reviews per application). Higher values indicate greater inter-rater reliability.

## Discussion

This study analyzed nearly 100,000 reviews across four Norwegian funders to investigate factors associated with reviewer disagreement in the evaluation of grant applications. Using the average deviation (AD) index as the outcome variable and a multilevel linear model to account for data structure, we found that several reviewer-, application-, and process-level variables were significantly associated with disagreement. While many effects were statistically significant due to the large sample size, most were small in absolute magnitude. Across predictors, the largest association, the expert-review format, was about 15% of the mean AD index, and the rest were smaller; for the review of an individual application these are small effects. Whether any is practically consequential depends on the end user, as anticipated in our inference criteria, since a factor that is minor for a single review may still matter to a funder or to society.

Additionally, while some effects were in line with our preregistered predictions, many of them were not supported by the analyses. Of the 15 predictions made, four were supported by our findings, seven did not show significant results and four showed significant results in the opposite direction of the prediction. Nevertheless, the findings offer valuable insights into the nature and determinants of reviewer disagreement in grant peer review.

### Individual-level sources

Two individual-level predictions were supported, both modestly: panels with more same-gender reviewers showed slightly lower disagreement, and panels with greater age heterogeneity slightly higher, consistent with demographic similarity supporting more aligned assessments. The proportion of young reviewers was unrelated to disagreement, unlike Seeber and colleagues, who found marginally higher disagreement among older reviewers and lower disagreement with program-specific experience [26]; because age proxies experience only imperfectly, we read this null cautiously (see Limitations).

The clearest individual-level result ran against prediction: expert reviews, in which reviewers held specialist knowledge of the application’s field, were associated with a 15% increase in disagreement rather than the decrease we expected. Greater expertise may heighten awareness of methodological and theoretical issues over which informed reviewers legitimately differ. Because expert reviews may also concentrate on the most complex applications, higher agreement would not necessarily mean higher validity.

### Structural-level sources

Research area showed the clearest structural pattern: disagreement was highest in the humanities and lowest in mathematics and the natural sciences, with the other fields between. This partly contrasts with prior work using the ICC, where the humanities showed the highest reliability [10]; the divergence likely reflects the different outcome metric (the AD index versus the ICC) and differences in evaluation procedures and reviewer pools, and is partly shaped by the data structure (see Limitations). Requested amount was unrelated to disagreement, echoing the negligible association Mutz et al. reported for reliability. Programs with greater variability in application amounts showed slightly lower disagreement, a small but reliable and previously unexamined association; a wider spread may make scope and fit more salient, or may proxy other program features.

Two process features pointed in opposite directions. In-depth reviews, involving more reviewer time and effort, were associated with a 6% reduction in disagreement, plausibly through more thorough engagement and more systematic use of criteria, while submission year showed a 12% reduction over ten years, consistent with gradual structural learning such as better matching and calibration. Taken with the expert-review result, this suggests that reviewer time and effort, not subject-matter expertise, are the more dependable lever for reducing disagreement.

### Theoretical framework: Reliability, noise, and the limits of evaluative diversity

The two distinctions introduced in the Introduction organise our reading of the variance decomposition. Level noise, the stable tendency of some reviewers to score systematically higher or lower, is an individual-level source. Pattern and occasion noise are not stable properties of reviewers: they arise when a particular reviewer meets a particular application. As the next subsection shows, disagreement in our data concentrates there, not at the reviewer level.

Two implications follow. First, reliability is a property of the measurement system, not of the individual judgments within it [34]: a process composed of individually well-reasoned reviews can still produce unstable funding decisions if the reviews are sufficiently dispersed. Second, some disagreement is functional, reflecting complementary expertise and legitimate evaluative diversity [35,36], and expert disagreement is the norm across judgment domains [37]. The question for funders is therefore not whether reviewers disagree, but whether decisions are stable; we return to this under Agreement and reliability.

### Reviewer and application heterogeneity

Reviewer-level heterogeneity accounts for just 3.1 percent of the total variance, compared with 24.1 percent at the application level and 55.2 percent at the residual (within-application) level. Program-level differences are negligible (0.5 percent), and funder-level differences—while larger at 17 percent—partly reflect the structure of the data, since three of the four funders operate only a single program. Taken together, these results indicate that most of the observed disagreement is not driven by persistent reviewer traits such as scoring style, strictness, or leniency. Instead, it largely reflects application-specific ambiguity and idiosyncratic variation in how individual criteria are interpreted when applied to particular proposals. Because such reviewer-by-application interactions are not stable reviewer traits, they cannot register at the reviewer level; they surface as application-level variance (when an application draws broadly divergent assessments) or in the residual (when the divergence is idiosyncratic to a particular reviewer-application pairing).

Because so little of the variance is attributable to stable reviewer-level differences, interventions aimed at “fixing” reviewers, such as calibration exercises, bias-reduction training, or restructuring reviewer pools, are unlikely on their own to reduce disagreement substantially, though they may still serve fairness, transparency, or perceived legitimacy. Larger gains are more likely to come at the application-reviewer interface, through clearer criteria, structured review formats, better anchoring examples, or more time per review, although even these may be incremental, since more than half of the variance is residual, reflecting genuine ambiguity and unavoidable noise in human judgment.

### Agreement and reliability

Although all four funders examined showed average intraclass correlation coefficients (ICC) that fall within the higher end of what has been documented in previous studies [38,39], they remain below the conventional threshold of 0.75 typically considered to reflect “good” reliability [7]. Specifically, our estimates of ICC(1,k), which reflect the reliability of the average score across the actual number of reviewers per application, ranged from 0.50 (Dam, NRC) to 0.65 (Cancer Society). These values imply that, in order to reach the threshold of 0.75, funders would require between 8 (Cancer Society) and 15 (Dam) independent reviewers per application, assuming similar score variability. These numbers are in line with other research on grant peer review [40] but still far beyond what is practically feasible for most grant review systems.

The largest funder contrast (Kavli highest mean AD, Dam lowest) should be read with caution: Kavli rests on a pool of only twelve reviewers, and an exploratory refit excluding it lowers the funder-level variance component from 17.0% to 0.5%, so the funder differences are almost entirely a Kavli small-pool artefact.

Relatedly, Dam’s combination of the lowest AD (0.54) and the lowest ICC(1,1) (0.18) is not a contradiction: AD measures within-application disagreement, whereas ICC is the share of variance lying between applications. Dam’s scores have the narrowest spread of the four funders (SD 0.84), so its between-application signal is compressed and reliability is depressed even though absolute agreement is high. This is the classical range-restriction effect on ICC [41,42].

As noted in the introduction, some degree of reviewer disagreement is both expected and potentially beneficial. Diverse perspectives may enrich the evaluative process and help identify blind spots in individual assessments [35,36].

The problem arises when disagreement is large enough to make outcomes unstable. If a plausible reassignment of reviewers would likely reverse a funding decision, the process lacks reliability and, by extension, legitimacy, and this holds whether the low reliability reflects range restriction, legitimate evaluative diversity, or noise. In that regime, *who* reviews the application matters more than *what* is being reviewed. This is an unacceptable scenario for any system aspiring to distribute research funding in a fair and evidence-based manner. The goal should not be to eliminate differences of opinion, but to keep them within bounds through clearer criteria, calibration, and sufficient averaging over independent reviews. To get there, funders first need to measure or estimate the reliability of their processes.

A common critique is that reliability estimates based on independent, individual reviews underestimate the “true” reliability of panel decisions, because panels typically discuss and agree on a single score. Two clarifications are important:

1. A single panel score masks within-panel dispersion: with only one consensus value per application, one cannot assess whether agreement was high or low.
2. Higher agreement after discussion does not necessarily reflect higher measurement reliability; it can also arise from social-influence dynamics (e.g., groupthink, authority, anchoring) that compress variation without increasing signal [43].

The most direct way to estimate the reliability of panel decisions would be to send replicate sets of applications to independent panels and compare outcomes, but this is usually both too expensive and practically difficult. In that context, independent individual reviews provide the best available basis for reliability estimation. Reliability measures – like the intraclass correlation – based on independent individual reviews are transparent and easily computable proxies for what panels aim to achieve by averaging information.

### Limitations

Several limitations qualify these findings. The data come from four Norwegian funders and may not generalize to other evaluation cultures or applicant populations, and the observational design identifies associations rather than causal mechanisms. The AD index captures the magnitude of disagreement but not its source, so it cannot separate justified from unjustified divergence. Missing data are a further concern: the excluded rows came almost entirely from the Research Council of Norway, so the missingness is not plausibly at random, and although our preregistered sensitivity analyses were largely consistent with the complete-case model, they only partly mitigate this.

The experience proxy also warrants caution. We used reviewer age because no direct measure of reviewing experience was available, but age also captures generational cohort, which cross-sectional data cannot disentangle from experience; the null result for proportion of young reviewers (IL2) is therefore consistent with no experience effect, an effect offset by a cohort effect, or a proxy too coarse to detect either.

Finally, two features of the funder structure limit the higher-level contrasts. Process characteristics were only partly standardized and partly unrecorded (calibration procedures, anchoring examples, reviewer training), and reviewer assignment is not random but matched to competence in funder-specific ways our data do not capture; both may contribute to the funder-level differences our data cannot fully resolve. The disciplinary composition is also uneven: the Research Council of Norway supplies 84% of the sample and nearly all cross-disciplinary variation, so the research-area contrasts are effectively within-NRC, and the humanities result reflects how NRC’s humanities panels operate rather than the humanities at large.

### Implications and future research

Reviewer disagreement remains a substantial challenge for the reliability of grant peer review: when it is large, funding outcomes depend too heavily on which reviewers an application happens to draw, threatening the legitimacy of peer review. Yet our results temper any expectation of an easy fix. The explained variance is low, and the variance structure itself bounds what interventions can achieve: funder, program, and reviewer effects together account for 20.6% of the variance in disagreement, against 24.1% between applications and 55.2% residual. Even if all funder, program, and reviewer variation were removed, overall disagreement would fall only modestly; normalizing every score within reviewers, which would eliminate all reviewer leniency and strictness, reduces disagreement by about 0.009 AD units, roughly 1.5%. The bulk reflects application-specific factors and idiosyncratic within-application variation in how criteria are read and weighted.

Improvements are therefore more likely to come from bundles of small changes at the application–reviewer interface, clearer criteria and rubrics, calibration and anchoring examples, structured review formats, better reviewer–application matching, and, where feasible, averaging over more independent reviews, than from any single lever. These measures should be evaluated rigorously, ideally with experimental or quasi-experimental designs, because the evidence base remains thin: a recent Cochrane review identified only ten controlled trials of reviewer training across grant and journal peer review and found no clear evidence of effective methods [44]. The pattern fits Kahneman et al’s observation that “wherever there is judgment, there is noise – and more of it than you think.” [15]; single-lever fixes are rare. For applicants, the same finding cuts one way: because much of the disagreement is interpretational, a proposal that states its aims and maps them explicitly onto the published criteria will likely give reviewers less room to diverge.

Future work would benefit from larger, multi-funder datasets with richer reviewer characteristics (e.g., expertise, workload) and process features (e.g., scoring systems, feedback), and from mixed-methods designs linking these to qualitative insight into how reviewers judge. Such work would sharpen the theory of judgment in peer review and give funders better-evidenced tools for making decisions more consistent and trustworthy.

## Supporting information

S1 Table Codebook for primary data set. Codebook describing the data in the primary data set used as the starting point for the analysis script (see S2 File).
(XLSX)

S2 Table Data exclusion. This table reports the number of cases excluded in the eight different steps of the data exclusion process described under the section “Analysis plan”.
(DOCX)

S3 Table Sensitivity analyses. This table reports results from sensitivity analyses described in the main text under the “Sensitivity analyses” subsection. Each model includes only one explanatory variable and uses all available data for that variable (i.e., without excluding rows with missing data on other predictors).
(DOCX)

S1 File Preprocessing script. This script has been used for producing the primary data set used as the basis for the analysis script (see S2 File). This includes merging the data sets from the four funders and producing the explanatory variables based on variables that could be used to identify reviewers or applications (gender and birth year). Additionally, all application IDs and reviewer IDs are switched with anonymized IDs.
(TXT)

S2 File Analysis script. This script describes the data exclusion, the production of the response variable, the remaining explanatory variables, removing of missing data, main analysis, sensitivity analyses and the production of the final codebook.
(TXT)

S1 Fig Forest plots of pairwise contrasts between research areas. A forest plot with pairwise comparisons between the seven categories of research area.
(PNG)

S2 Fig AD index by year. A line plot showing the AD index with error bars per year from 2013 to 2024.
(PNG)
