# Supplementary material for: Why do reviewers disagree? Evidence from four funders and 134,000 reviews
Source: PLoS One. 2026 Sep 23;21(9):e0356108. doi: 10.1371/journal.pone.0356108 (PMC13600531; doi:10.1371/journal.pone.0356108)
Supplement: Supplementary file 1 [file pone.0356108.s002.docx]

**S2 Table. Number of rows removed at each step of the data exclusion process**

This table reports the number of cases excluded in the eight different steps of the data exclusion process described under the section “Analysis plan”.

| Step | Criterion | Rows Removed | Rows Remaining |
| --- | --- | --- | --- |
| 1 | Unlikely reviewer ages (under 21 or over 100) | 4 | 134,987 |
| 2 | Duplicate entries | 81 | 134,906 |
| 3 | Missing review scores | 4,549 | 130,357 |
| 4 | Impossible review scores (<1 or >7) | 0 | 130,357 |
| 5 | Missing application amounts | 21 | 130,336 |
| 6 | Unlikely application amounts (>10× program average) | 7 | 130,329 |
| 7 | Fewer than two reviews of a given type per application | 32,137 | 98,192 |
| 8 | Programs with fewer than 20 applications | 518 | 97,674 |
