# Supplementary material for: Why do reviewers disagree? Evidence from four funders and 134,000 reviews
Source: PLoS One. 2026 Sep 23;21(9):e0356108. doi: 10.1371/journal.pone.0356108 (PMC13600531; doi:10.1371/journal.pone.0356108)
Supplement: Supplementary file 2 [file pone.0356108.s003.docx]

**S3 Table.** Sensitivity analyses regressing each explanatory variable separately on reviewer disagreement.

This table reports results from sensitivity analyses described in the main text under the “Sensitivity analyses” subsection. Each model includes only one explanatory variable and uses all available data for that variable (i.e., without excluding rows with missing data on other predictors). The final column indicates whether the direction and statistical significance of the result matches the main model presented in Table 5. Variables are labelled as in the manuscript; they were renamed relative to the Stage 1 protocol (the mapping to the protocol’s variable labels is given under Changes to the registered report protocol in the manuscript).

|  | Sensitivity analyses | | | | Main model | |  |
| --- | --- | --- | --- | --- | --- | --- | --- |
| Variable | N observations | β | p-value | R² | β | p-value | Consistency with main model |
| IL1a Proportion same gender | 96,029 | -0.033 | <0.001 | 0.000 | -0.023 | 0.012 | Match |
| IL1b SD reviewer age | 89,442 | 0.003 | <0.001 | 0.001 | 0.002 | <0.001 | Match |
| IL2 Proportion young reviewers | 89,442 | 0.021 | 0.001 | 0.000 | -0.001 | 0.871 | Diff (direction & significance) |
| IL3 Expert review | 97,674 | 0.073 | 0.000 | 0.001 | 0.087 | <0.001 | Match |
| SL1 Funding amount (MNOK) | 97,674 | 0.000 | 0.127 | 0.000 | -0.001 | 0.057 | Match (ns) |
| SL2 Variability in application amount | 97,674 | 0.018 | 0.227 | 0.000 | -0.026 | 0.306 | Match (ns) |
| SL3 In-depth review | 97,674 | -0.027 | 0.000 | 0.001 | -0.034 | <0.001 | Match |
| SL4a Humanities | 97,643 | 0.037 | 0.000 | 0.000 | 0.032 | 0.001 | Match |
| SL4b Social sciences | 97,643 | 0.018 | 0.000 | 0.000 | 0.013 | 0.077 | Diff (significance only) |
| SL4c Technology | 97,643 | -0.004 | 0.300 | 0.000 | 0.015 | 0.033 | Diff (direction & significance) |
| SL4d Medicine & health sciences | 97,643 | 0.046 | <0.001 | 0.002 | 0.034 | <0.001 | Match |
| SL4e Mathematics & natural sciences | 97,643 | -0.081 | <0.001 | 0.006 | -0.043 | <0.001 | Match |
| SL4f Agriculture & fisheries | 97,643 | -0.021 | 0.002 | 0.000 | -0.014 | 0.214 | Diff (significance only) |
| SL4g Other disciplines | 97,643 | -0.568 | 0.084 | 0.000 | — | — | n/a (ref. in main model) |
| SL5 Year of submission | 97,674 | -0.006 | 0.000 | 0.001 | -0.007 | <0.001 | Match |

Note: β = unstandardized coefficient. R² values are from separate bivariate models and are small for all predictors (≤ 0.006). Consistency categories: ‘Match’ = same direction and significance; ‘Match (ns)’ = non-significant in both models; ‘Diff (significance only)’ = same direction but differs in significance; ‘Diff (direction only)’ = differs in direction; ‘Diff (direction & significance)’ = differs in both. EV3g served as the reference category in the main model.
