# Supplementary material for: Why do reviewers disagree? Evidence from four funders and 134,000 reviews
Source: PLoS One. 2026 Sep 23;21(9):e0356108. doi: 10.1371/journal.pone.0356108 (PMC13600531; doi:10.1371/journal.pone.0356108)
Supplement: Peer review history

# Peer review: Decision Letter 0

**DOI:** 10.1371/journal.pone.0356108.r001

**Authors:** Tord Ivarsson

1 Apr 2026

PONE-D-25-67755

Why do reviewers disagree? Evidence from four funders and 134,000 reviews

PLOS One

Dear Dr. Hesselberg,

Thank you for submitting your manuscript to PLOS ONE. After careful consideration, we feel that it has merit but does not fully meet PLOS ONE’s publication criteria as it currently stands. Therefore, we invite you to submit a revised version of the manuscript that addresses the points raised during the review process.

Please submit your revised manuscript by May 16 2026 11:59PM. If you will need more time than this to complete your revisions, please reply to this message or contact the journal office at plosone@plos.org. When you're ready to submit your revision, log on to https://www.editorialmanager.com/pone/ and select the 'Submissions Needing Revision' folder to locate your manuscript file.

Please include the following items when submitting your revised manuscript:

- A letter that responds to each point raised by the academic editor and reviewer(s). You should upload this letter as a separate file labeled 'Response to Reviewers'.
- A marked-up copy of your manuscript that highlights changes made to the original version. You should upload this as a separate file labeled 'Revised Manuscript with Track Changes'.
- An unmarked version of your revised paper without tracked changes. You should upload this as a separate file labeled 'Manuscript'.

If you would like to make changes to your financial disclosure, please include your updated statement in your cover letter. Guidelines for resubmitting your figure files are available below the reviewer comments at the end of this letter.

If applicable, we recommend that you deposit your laboratory protocols in protocols.io to enhance the reproducibility of your results. Protocols.io assigns your protocol its own identifier (DOI) so that it can be cited independently in the future. For instructions see: https://journals.plos.org/plosone/s/submission-guidelines#loc-laboratory-protocols. Additionally, PLOS ONE offers an option for publishing peer-reviewed Lab Protocol articles, which describe protocols hosted on protocols.io. Read more information on sharing protocols at https://plos.org/protocols?utm_medium=editorial-email&utm_source=authorletters&utm_campaign=protocols.

As the corresponding author, your ORCID iD is verified in the submission system and will appear in the published article. PLOS supports the use of ORCID, and we encourage all coauthors to register for an ORCID iD and use it as well. Please encourage your coauthors to verify their ORCID iD within the submission system before final acceptance, as unverified ORCID iDs will not appear in the published article. *Only*  the individual author can complete the verification step; PLOS staff *cannot*  verify ORCID iDs on behalf of authors.

We look forward to receiving your revised manuscript.

Kind regards,

Tord Ivarsson, MD, PhD

Academic Editor

PLOS One

**Journal Requirements:**

When submitting your revision, we need you to address these additional requirements.

1. Please ensure that your manuscript meets PLOS ONE’s style requirements, including those for file naming. The PLOS ONE style templates can be found at

https://journals.plos.org/plosone/s/file?id=wjVg/PLOSOne_formatting_sample_main_body.pdf and

https://journals.plos.org/plosone/s/file?id=ba62/PLOSOne_formatting_sample_title_authors_affiliations.pdf

2. In your cover letter, please confirm that the research you have described in your manuscript, including participant recruitment, data collection, modification, or processing, has not started and will not start until after your paper has been accepted to the journal (assuming data need to be collected or participants recruited specifically for your study). In order to proceed with your submission, you must provide confirmation.

3. Thank you for stating the following financial disclosure:

“Funds provided by Stiftelsen Dam.”

Please state what role the funders took in the study.  If the funders had no role, please state: "The funders had no role in study design, data collection and analysis, decision to publish, or preparation of the manuscript."

If this statement is not correct you must amend it as needed.

Please include this amended Role of Funder statement in your cover letter; we will change the online submission form on your behalf.

4. Please note that funding information should not appear in any section or other areas of your manuscript. We will only publish funding information present in the Funding Statement section of the online submission form. Please remove any funding-related text from the manuscript.

5. Please ensure that you refer to Figure 1 in your text as, if accepted, production will need this reference to link the reader to the figure.

6. If the reviewer comments include a recommendation to cite specific previously published works, please review and evaluate these publications to determine whether they are relevant and should be cited. There is no requirement to cite these works unless the editor has indicated otherwise.

7. Please review your reference list to ensure that it is complete and correct. If you have cited papers that have been retracted, please include the rationale for doing so in the manuscript text, or remove these references and replace them with relevant current references. Any changes to the reference list should be mentioned in the rebuttal letter that accompanies your revised manuscript. If you need to cite a retracted article, indicate the article’s retracted status in the References list and also include a citation and full reference for the retraction notice.

**Additional Editor Comments:**

The study has many strengths but also some shortcomings, and how these should be viewed leads one reviewer to recommend a major revision and the second to recommend accepting the submission, albeit with some pertinent recommendation to changes. My view is that a major revision is not really needed as the empirical part with data acquisition and analysis as well as the way you report results are good. However, a minor revision is on the slight side as the recommendation reviewer 1, as well as reviewer 2 has, include recommendations that require a good deal of work.

The reviews obtained come from two different perspectives: first that of the senior researcher in the field of humanitarian studies; and the second that of a researcher familiar with this type of study. In addition, I as academic editor and with an experience of getting both positive and negative responses to grant applications will add a few aspects.

First, reviewer 1 points out a need for a review of the theoretical basis of the introduction – and discussion. I agree and formed the impression that the limitations to the data that were available have had an impact on the theoretical framework. What types of data that are lacking should be pointed out in the limitations section. Could funders provide more in-depth data?

Going beyond those limitations may be fruitful when discussing the outcome. If you do not agree with the need for a more coherent theoretical framework, your argument needs to be clear and concise.

Also, reviewer 1 points out that “reviewer agreement/disagreement” operationalization needs a stricter rationale. I understand that the data available sets some limits but would anyway like to see your response to this aspect in a revised form.

When responding, ascertain that you have covered all points reviewer 1 provides.

When doing this, ascertain that you abbreviate both sections substantially, as reviewer 1 as well points out. The text is too long for the ordinary researcher browsing the PLOS. If you wish to reach a wider audience, the message needs to be easier to take in.

Reviewer 2 has many more technical recommendations that show familiarity with this research field that neither I nor reviewer 1 has. When you respond to those points, be sure to clarify your position to the recommendations in your response so that I, in a next step of the editing process, can understand the rationales of your response.

As a senior researcher myself, I think that your paper should end up with some pointers to guidelines for better grant applications. As now you just state the need, but your data and a developed theoretical framework should render some concrete points that could be helpful to both grant providers and grant application writers. Should the review of the empirical methods to be used in the research proposed be judged independently of the theoretical basis of the research question/s? An impression I have had and as well heard from colleagues is that some grant reviewers will judge the methodology based on their view of the research question. How can this be handled in the review process?

So, my summary conclusion is that a “minor revision”, which I recommend, needs to be quite substantial and close to a major revision.

[Note: HTML markup is below. Please do not edit.]

Reviewers' comments:

Reviewer’s Responses to Questions

**Comments to the Author**

1. Does the manuscript adhere to the experimental procedures and analyses described in the Registered Report Protocol?

If the manuscript reports any deviations from the planned experimental procedures and analyses, those must be reasonable and adequately justified.

Reviewer #1: Yes

Reviewer #2: Yes

**********

2. If the manuscript reports exploratory analyses or experimental procedures not outlined in the original Registered Report Protocol, are these reasonable, justified and methodologically sound?

A Registered Report may include valid exploratory analyses not previously outlined in the Registered Report Protocol, as long as they are described as such.

Reviewer #1: Yes

Reviewer #2: Yes

**********

3. Are the conclusions supported by the data and do they address the research question presented in the Registered Report Protocol?

The manuscript must describe a technically sound piece of scientific research with data that supports the conclusions. The conclusions must be drawn appropriately based on the research question(s) outlined in the Registered Report Protocol and on the data presented.

Reviewer #1: Partly

Reviewer #2: Yes

**********

4. Have the authors made all data underlying the findings in their manuscript fully available?

The PLOS Data policy requires authors to make all data underlying the findings described in their manuscript fully available without restriction, with rare exception (please refer to the Data Availability Statement in the manuscript PDF file). The data should be provided as part of the manuscript or its supporting information, or deposited to a public repository. For example, in addition to summary statistics, the data points behind means, medians and variance measures should be available. If there are restrictions on publicly sharing data—e.g. participant privacy or use of data from a third party—those must be specified.

Reviewer #1: Yes

Reviewer #2: Yes

**********

5. Is the manuscript presented in an intelligible fashion and written in standard English?

*PLOS ONE*  does not copyedit accepted manuscripts, so the language in submitted articles must be clear, correct, and unambiguous. Any typographical or grammatical errors should be corrected at revision, so please note any specific errors here.

Reviewer #1: Yes

Reviewer #2: Yes

**********

6. Review Comments to the Author

Please use the space provided to explain your answers to the questions above. (Please upload your review as an attachment if it exceeds 20,000 characters)

Reviewer #1: This is a robust analysis on factors affecting reviewer disagreement, based on a large number of peer reviews in the purpose of funding. As such, the paper can make an important contribution to a large range of readership. However, certain issues ought to be clarified/addressed.

- Please, provide a coherent conceptual framework for the different sources of potential reviewer disagreement, considering for instance individual versus structural sources and their interactions. For instance, reviewer experience and expertise are currently handled as independent, individual features, but are reviewers assigned at random? Yet, could assignment procedures themselves (random, or matching experience to application complexity, etc), be seen as an antecedent of disagreement?

- A coherent theoretical structure will help guide a more comprehensive and balanced discussion of limitations and implications, and secure the added value of the discussion - regarding the importance of structural competence and how to promote it, both at an individual and at a procedural level, but also regarding how to understand findings in the context of the theoretical and empirical knowledge regarding expert judgment processes and processes of decisions under uncertainty.

- Please explain more clearly how reviewer agreement/disagreement was operationalized, and why this measure is used instead of a rater internal consistency index. Please, explain also the range for the used index and help the reader understand reported difference sizes.

- Specificity and operationalization of assessment criteria are neither operationalized nor measured. This, despite the fact that they comprise a central feature of the review process, as also discussed in conclusions and recommendations. Please, justify this choice and its implictions/limitations.

- Two additional limitations concern (a) the operationalization of experience of reviewer (age could be more indicative of specific cohort effects than of actual experience, and in the particular analysis a confounding) and (b) the deletion of cases with missing data. Please, discuss sufficiently.

- Although the dataset is large, to my understanding, three of the four funders operate within the domain of medicine/health. The manuscript should more explicitly acknowledge this imbalance and its implications for generalizability and discussions regarding disagreement differences across disciplines.

- Most effects are small, as the authors also note, yet the manuscript still interprets these findings substantively. A stricter distinction between statistical significance and practical relevance is needed throughout, especially in Results and Discussion. Consider systematically contextualizing all effect sizes relative to the mean AD index to reinforce their limited magnitude.

- The manuscript is currently long and somewhat repetitive. Please, review and revise, to avoid repetition.

Thank you for trusting me with this review.

Reviewer #2: Why do reviewers disagree? Evidence from four funders and 134,000 reviews

This registered report analyses reviewer scoring of grant applications, across four Norwegian research funders, looking at predictors of reviewer disagreement. Although modest predictors are found among reviewer characteristics, far more variability in disagreement was attributable to the application-level and residual variability. This supports the idea that reviewer disagreement is more due to ambiguity (around the application and/or review criteria) rather than factors such as reviewer leniency. The report is supported by anonymised open data and open analysis code.

Overall this is a thoroughly and clearly presented report which makes a substantial contribution.

I have some comments, none of which should stand in the way of publication:

- It seems that Dam is getting reviewer disagreement comparable to NRC or Cancer society, despite having review volumes like Kavli (many reviewers per reviewer, many applications per reviewer). Does that reveal something about the Dam review process (this in an invitation to speculate, I recognise that the data cannot definitively arbitrate on this)

- That said, the ICCs reported in Table 6 put Dam as lowest - is there a contradiction with the by-funder AD scores (e.g. Figure 3 and Table 3)

- It is important to recognise, when assessing reviewer (dis)agreement that range restriction will decrease the ability to detect reviewer consistency (inflating the role of noise). i.e. if a funder only sees high quality applications and/or sends the best applications for review, then the "task" of reviewers being consistent with each other is harder (Boudreau, 2026; Erosheva et al, 2021). I think this applies statistically to ICC (but maybe not to the AD statistic, the authors can comment if they know), but also "psychologically" since reviewers will tent to calibrate their judgement to make discriminations over the range of quality they expect to see.

- The measure of reviewing experience seems pretty arbitrary. Were alternative specifications explored?

- You report (Table 5) that more variance is explained by applications than reviewers. Your regression is predicting Reviewer Disagreement, so not score directly, but the variance in scores awarded, if I read this right? So is this interpretation compatible, or even suggested, by your analysis: reviewer disagreement is not predicted by reviewer bias (in the sense of leniency or not), hence the low amount of variance attributable to reviewer. Instead, disagreement may arise from interpretational ambiguity - how different reviewers interpret the scoring criteria as applying to that application. If we were predicting score this would be an interaction effect (application * reviewer), but because you use disagreement as the predictor I am not sure if it would appear as variance from applications or in the residual. What do you think?

References

Boudreau, K. (2026). Field Experiments in the Science of Science: Lessons from Peer Review and the Evaluation of New Knowledge (No. w34811). National Bureau of Economic Research.

Erosheva, E. A., Martinková, P., & Lee, C. J. (2021). When zero may not be zero: A cautionary note on the use of inter‐rater reliability in evaluating grant peer review. Journal of the Royal Statistical Society: Series A (Statistics in Society), 184(3), 904-919

**********

7. PLOS authors have the option to publish the peer review history of their article (what does this mean?). If published, this will include your full peer review and any attached files.

If you choose “no”, your identity will remain anonymous but your review may still be made public.

**Do you want your identity to be public for this peer review?**  For information about this choice, including consent withdrawal, please see our Privacy Policy.

Reviewer #1: No

Reviewer #2: **Yes:** Tom Stafford

**********

[NOTE: If reviewer comments were submitted as an attachment file, they will be attached to this email and accessible via the submission site. Please log into your account, locate the manuscript record, and check for the action link "View Attachments". If this link does not appear, there are no attachment files.]

To ensure your figures meet our technical requirements, please review our figure guidelines: https://journals.plos.org/plosone/s/figures

You may also use PLOS’s free figure tool, NAAS, to help you prepare publication quality figures: https://journals.plos.org/plosone/s/figures#loc-tools-for-figure-preparation.

NAAS will assess whether your figures meet our technical requirements by comparing each figure against our figure specifications.

# Author comment: Author response to Decision Letter 1

**DOI:** 10.1371/journal.pone.0356108.r002

3 Jul 2026

We are grateful to the Editor and both reviewers for the careful and constructive engagement with the manuscript. The revision is stronger for it, and several of the points raised — particularly around the theoretical framework, length, and the interpretation of effect sizes — pointed to things we should have addressed more clearly in the originally submitted version. We have tried throughout the revision to address each point on its own merits first.

A short note on how the Stage 2 framing has shaped some of our responses, in case it is helpful to the Editor in reading the rest of this letter. The manuscript is a Stage 2 submission against a Stage 1 protocol that received in-principle acceptance and was published in May 2025 (Hesselberg et al., 2025). Consistent with PLOS ONE’s published Registered Reports guidance — that peer review at Stage 2 focuses on adherence to the protocol, and that the journal commits to publishing a study if the accepted protocol is followed — both reviewers answered "Yes" to the gate question on protocol adherence.

In practice this has meant that, for a small subset of reviewer concerns — those that touch the preregistered hypotheses, the response variable, the explanatory variables and their operationalisation, the exclusion criteria, or the main model specification — we have preserved the preregistered choice and added new material around it (clarifications, additional context in the Methods, sharpened limitations, and where appropriate exploratory analyses, which the PLOS ONE guidelines explicitly permit). For the majority of the points raised — the Introduction’s framing, the Discussion, the Limitations, the Implications, effect-size interpretation, length, and communication quality — we have made substantive changes. We have tried to make this distinction visible in our individual responses below, not as a defensive move, but to make the rationale for each response transparent.

RESPONSE TO EDITOR

E1. Theoretical basis of the Introduction and Discussion; what data are lacking

Editor: "Reviewer 1 points out a need for a review of the theoretical basis of the introduction – and discussion. ... What types of data that are lacking should be pointed out in the limitations section."

Response.

Both halves are addressed. The theoretical basis is now explicit: as set out under R1.1, the research questions are regrouped into individual-level and structural sources, the Introduction adds a framing paragraph and the noise taxonomy, and a new Discussion subsection (Theoretical framework, see R1.2) applies the distinction to the variance components. The data that are lacking are now named in Limitations: reviewer-assignment procedures (R1.1), the specificity of each funder’s criteria (R1.4), the experience proxy and its cohort confound (R1.5a), and the disciplinary imbalance across funders (R1.6).

Manuscript changes: as detailed under R1.1, R1.2, R1.4, R1.5a, and R1.6.

E2. Operationalisation rationale for reviewer agreement/disagreement

Editor: "Reviewer 1 points out that 'reviewer agreement/disagreement' operationalization needs a stricter rationale."

Response

Addressed under R1.3, where we expand the Methods rationale for the AD index, including why we use it rather than a rater-consistency coefficient such as the ICC. The conceptual distinction between agreement (AD) and reliability (ICC), and why they can diverge, is developed under R2.2 and R2.3.

Manuscript changes: as detailed under R1.3 (Methods) and R2.2 (Discussion).

E3. Length: abbreviate Introduction and Discussion substantially

Editor: "...abbreviate both sections substantially..."

Response.

We agree, and both sections are now substantially shorter and less repetitive. Measured against the version you reviewed, the Introduction is down 28% (2,138 to 1,548 words) and the Discussion 9% (2,372 to 2,149), with the manuscript about 600 words shorter overall. The Discussion figure understates the compression: it now also carries several passages the reviews required (the theoretical framework, the AD-versus-ICC and range-restriction analysis, the exploratory Kavli refit, and two added limitations), so a net 9% reduction means the original text was cut considerably more.

The cuts targeted the repetition you and Reviewer 1 identified. In the Discussion, five topical subsections that restated the Results predictor by predictor were replaced by two compact subsections organised around the framework (Individual-level and Structural-level sources); the variance-decomposition material was consolidated; and the closing implications, which had made their central point three times, were reduced to one. In the Introduction, the enumerated lists of reliability coefficients and of the Austrian study’s predictors were condensed to their essentials. Throughout, the goal was to make the argument easier for a general reader to take in, as you suggest. This also addresses Reviewer 1’s related point (R1.8).

E4. Clarify our position on each of Reviewer 2’s technical points

Response. Our position on each of Reviewer 2’s points is set out under R2.1 to R2.5: the Dam-versus-Kavli funder contrast, with an exploratory Kavli-excluded refit showing the funder-level signal is almost entirely Kavli’s small reviewer pool (R2.1); the reconciliation of the by-funder AD and ICC values through range restriction (R2.2 and R2.3); the rationale for the experience measure and the alternative specification we explored (R2.4); and the reviewer-by-application reading of the residual variance, which we adopt from Reviewer 2 (R2.5).

E5. Concrete pointers and guidelines for better grant review processes

Editor: "...your paper should end up with some pointers to guidelines... As now you just state the need..."

Response. We have taken this up, and it now runs through the revised Implications section rather than a bare statement of need. The concrete levers follow from the individual-versus-structural framework (E1) and from the variance decomposition. For funders, they sit at the application–reviewer interface: clearer criteria and rubrics, calibration and anchoring examples, structured review formats, better reviewer–application matching, and, where reliability is critical, averaging over more independent reviews, the one route that can reach conventional reliability, at a throughput cost we make explicit (roughly 8 to 15 reviewers per application for ICC 0.75). For applicants, our central finding, that most disagreement is interpretational and application-level rather than reviewer-driven, implies that a proposal stating its aims and mapping them onto the published criteria gives reviewers less room to diverge. We deliberately stop short of stronger guidelines: with an observational design and most variance residual, no single change can be promised to raise reliability substantially, and we state this rather than overreach.

E6. Independent judgment of methods vs. research question

Editor: "Should the review of the empirical methods... be judged independently of the theoretical basis of the research question/s?"

Response. Our data bear on this, though they cannot settle it. The relevant result is the expert-review finding (Individual-level sources): reviewers with specialist knowledge of an application’s field disagreed more, not less, the largest single effect we observed and contrary to our prediction. One plausible reading is the entanglement the editor describes, that deeper subject engagement brings the reviewer’s own view of the research question to bear and colors the assessment of the methods, widening divergence. The structural response this points to is to score the soundness of the proposed methods on its own dimension, separately from the appeal of the research question, so a reviewer’s view of the question is less free to drive the methods' score. We find this a credible direction, consistent with our general position that structural changes to the review format are the more promising levers. We are careful not to overstate it: our outcome is overall disagreement, not dimension-level scores, so the data motivate this idea rather than test it, and we offer it as a hypothesis for rigorous, ideally experimental, evaluation.

RESPONSES TO REVIEWER #1

R1.1. Coherent conceptual framework for sources of disagreement

Reviewer: "Please, provide a coherent conceptual framework for the different sources of potential reviewer disagreement, considering for instance individual versus structural sources and their interactions. For instance, reviewer experience and expertise are currently handled as independent, individual features, but are reviewers assigned at random? Yet, could assignment procedures themselves (random, or matching experience to application complexity, etc), be seen as an antecedent of disagreement?"

Response.

We thank the reviewer for this suggestion, which we have adopted as an organising principle for the revision. We agree with the reviewer that the framework was implicit: the eight research questions always concerned two kinds of sources, but the previously submitted manuscript neither said so nor structured its presentation around them.

The revised manuscript makes the framework explicit and load-bearing. The research questions and explanatory variables are regrouped and relabelled into individual-level sources, properties of the reviewers who assess an application (IL1 reviewer similarity = EV1, IL2 reviewing experience = EV2, IL3 specialist knowledge = EV7), and structural sources, properties of what is reviewed and how the review is organised (SL1 application amount = EV4, SL2 within-programme variability = EV5, SL3 review depth = EV6, SL4 research area = EV3, SL5 year of submission = EV8). The relabelling is documented under Changes to the protocol; operationalisations, hypotheses, predicted directions, and analyses are unchanged. The relabelling is documented under Changes to the protocol with the mapping to the protocol’s EV labels; operationalisations, hypotheses, predicted directions, and analyses are unchanged.

The revised Introduction introduces Kahneman, Sibony, and Sunstein’s noise taxonomy (level, pattern, and occasion noise) and the cross-cutting distinction between individual-level and structural sources of disagreement, and a short paragraph at the head of the research questions maps the eight questions onto the two levels and notes their interaction. A new Discussion subsection (see R1.2) applies the same distinction as the interpretive lens for the variance decomposition.

On assignment procedures specifically: we agree, and the reviewer’s framing of assignment as an antecedent of disagreement is exactly the kind of cross-level interaction the framework highlights. None of the four funders assigns reviewers at random; all match application topic to reviewer competence, differing in matching granularity, pool size, and whether a coordinator or an algorithm makes the assignment. The revised Sample section states this explicitly. Because no application-review-level variable captures the procedure, it could not be a preregistered predictor; the revised Limitations notes assignment procedures as an unmeasured antecedent and a candidate contributor to between-funder variance that our data cannot isolate.

Manuscript changes: research questions and variables regrouped and relabelled (IL/SL), documented under Changes to the protocol; new framing paragraph at the head of Research questions and predictions; matching-not-randomisation made explicit in Sample; new Limitations note on assignment procedures; new Theoretical framework subsection in the Discussion (see R1.2).

R1.2. Theoretical structure for limitations and implications

Reviewer: “A coherent theoretical structure will help guide a more comprehensive and balanced discussion of limitations and implications, and secure the added value of the discussion - regarding the importance of structural competence and how to promote it, both at an individual and at a procedural level, but also regarding how to understand findings in the context of the theoretical and empirical knowledge regarding expert judgment processes and processes of decisions under uncertainty.”

Response.

The new Theoretical framework subsection in the Discussion (see R1.1) provides this structure. It reads the variance decomposition through the noise taxonomy introduced in the Introduction and through classical test theory, on which reliability is a property of the measurement system rather than of the individual judgments within it (Lord & Novick, 1968), and it situates our results in the broader finding that expert disagreement is the norm across judgment domains (Hodgson, 2008). The same individual-versus-structural lens then guides the Limitations and the practical implications.

Manuscript changes: new Theoretical framework subsection in the Discussion (shared with R1.1).

R1.3. Operationalisation of reviewer agreement/disagreement

Reviewer: " Please explain more clearly how reviewer agreement/disagreement was operationalized, and why this measure is used instead of a rater internal consistency index. Please, explain also the range for the used index and help the reader understand reported difference sizes.”

Response.

Thank you for pointing this out; we have expanded the Methods rationale and address each part of the question here. The outcome is the within-application average deviation (AD) index: for each application, the mean absolute deviation of the individual reviewer scores from that application’s mean, in the original 1–7 scale units. It is zero when reviewers agree perfectly and grows as they diverge, with a maximum of 3 on a 1–7 scale (scores split between the extremes); in our data the mean is 0.570. We use it rather than a rater-consistency coefficient because it expresses disagreement directly in raw scale units, requires no assumed null distribution, and is robust to assumptions about reviewer calibration (Burke, Finkelstein & Dusig, 1999; Burke & Dunlap, 2002; Roberson, Sturman & Simons, 2007). A coefficient such as the ICC answers a different question, the share of total score variance lying between applications: it is a reliability rather than an agreement coefficient, and the two can diverge sharply when the range of application quality is compressed (range restriction). We therefore take the AD index as the primary outcome and report ICCs in exploratory analyses only, for benchmarking against prior work; the AD-versus-ICC distinction and its consequences are developed under R2.2 and R2.3. Finally, to help the reader gauge difference sizes, every effect in the Results is now expressed relative to the mean AD index (0.570), so its practical magnitude can be read directly in disagreement units; the largest, the expert-review format, is about 15% of the mean, and the rest are smaller (see R1.7).

Manuscript changes: expanded the AD-index rationale and stated its range in the Methods (Response variable), with fuller citations (Burke et al., 1999; Burke & Dunlap, 2002; Roberson et al., 2007); effects throughout the Results expressed relative to the mean AD index (see R1.7); the AD-versus-ICC distinction developed in the Discussion (see R2.2, R2.3).

R1.4. Specificity and operationalisation of assessment criteria

Reviewer: " Specificity and operationalization of assessment criteria are neither operationalized nor measured. This, despite the fact that they comprise a central feature of the review process, as also discussed in conclusions and recommendations. Please, justify this choice and its implictions/limitations."

Response.

Correct. Useful background: from 2019 onward, NRC, Kavli, and Stiftelsen Dam have used the same review criteria and the same 1–7 scale for the bulk of their evaluations; NCS uses a closely related set on the same scale. Arguably, the funders are not as heterogeneous in instrument design as they might appear post-2019.

Manuscript changes: We have included this in Methods and draw on it when reading cross-funder contrasts. Limitations acknowledge that calibration procedures, anchoring-example explicitness, and reviewer training were not separately measured.

R1.5a. Experience proxy — age and cohort e

# Editor report: Decision Letter 1

**DOI:** 10.1371/journal.pone.0356108.r003

**Authors:** Tord Ivarsson

29 Jul 2026

Why do reviewers disagree? Evidence from four funders and 134,000 reviews

PONE-D-25-67755R1

Dear Dr. Hesselberg,

We’re pleased to inform you that your manuscript has been judged scientifically suitable for publication and will be formally accepted for publication once it meets all outstanding technical requirements. I find your revisions based on both reviewers points to be acceptable. I appreciate that the data that are available through the grant organisations set the limits of what can be statistically analysed and examined. I sincerely hope that the grant bodies read your paper and provide better data for your future studies.

Within one week, you’ll receive an e-mail detailing the required amendments. When these have been addressed, you’ll receive a formal acceptance letter and your manuscript will be scheduled for publication.

An invoice will be generated when your article is formally accepted. Please note, if your institution has a publishing partnership with PLOS and your article meets the relevant criteria, all or part of your publication costs will be covered. Please make sure your user information is up-to-date by logging into Editorial Manager at Editorial Manager® and clicking the ‘Update My Information' link at the top of the page. For questions related to billing, please contact billing support.

If your institution or institutions have a press office, please notify them about your upcoming paper to help maximize its impact. If they’ll be preparing press materials, please inform our press team as soon as possible -- no later than 48 hours after receiving the formal acceptance. Your manuscript will remain under strict press embargo until 2 pm Eastern Time on the date of publication. For more information, please contact onepress@plos.org.

Kind regards,

Tord Ivarsson, MD, PhD

Academic Editor

PLOS One

# Editor report: Acceptance letter

**DOI:** 10.1371/journal.pone.0356108.r004

**Authors:** Tord Ivarsson

PONE-D-25-67755R1

PLOS One

Dear Dr. Hesselberg,

I'm pleased to inform you that your manuscript has been deemed suitable for publication in PLOS One. Congratulations! Your manuscript is now being handed over to our production team.

At this stage, our production department will prepare your paper for publication. This includes ensuring the following:

* All references, tables, and figures are properly cited

* All relevant supporting information is included in the manuscript submission,

* There are no issues that prevent the paper from being properly typeset

You will receive further instructions from the production team, including instructions on how to review your proof when it is ready. Please keep in mind that we are working through a large volume of accepted articles, so please give us a few days to review your paper and let you know the next and final steps.

Lastly, if your institution or institutions have a press office, please let them know about your upcoming paper now to help maximize its impact. If they'll be preparing press materials, please inform our press team within the next 48 hours. Your manuscript will remain under strict press embargo until 2 pm Eastern Time on the date of publication. For more information, please contact onepress@plos.org.

You will receive an invoice from PLOS for your publication fee after your manuscript has reached the completed accept phase. If you receive an email requesting payment before acceptance or for any other service, this may be a phishing scheme. Learn how to identify phishing emails and protect your accounts at https://explore.plos.org/phishing.

If we can help with anything else, please email us at customercare@plos.org.

Thank you for submitting your work to PLOS One and supporting open access.

Kind regards,

PLOS ONE Editorial Office Staff

on behalf of

Dr. Tord Ivarsson

Academic Editor

PLOS One
